# Linear and nonlinear relationship between chronological age and maturity status with handgrip strength in children and adolescents

**DOI:** 10.3389/fped.2025.1516798

**Published:** 2025-08-29

**Authors:** Marco Cossio-Bolaños, Rubén Vidal-Espinoza, Luis Felipe Castelli Correia de Campos, Enio Ricardo Vaz-Ronque, Evandro Lázari, José Fuentes-López, Miguel de Arruda, Jose Sulla-Torres, Rossana Gomez-Campos

**Affiliations:** ^1^ Departamento de Ciencias de la Actividad Física, Universidad Católica del Maule, Talca, Chile; ^2^ Universidad Católica Silva Henriquez, Santiago, Chile; ^3^ Núcleo de Investigación en Ciencias de la Motricidad Humana, Universidad Adventista de Chile, Chillán, Chile; ^4^ State University of Londrina, Londrina, Brazil; ^5^ Faculdade de Educação Física, Universidade Estadual de Campinas, Sao Paulo, Brazil; ^6^ Escuela Profesional de Educación Física, Universidad Nacional del Altiplano, Puno, Perú; ^7^ Universidad Católica de Santa María, Arequipa, Perú

**Keywords:** hand grip strength, age, maturation, children, adolescent, nonlinear modeling

## Abstract

**Introduction:**

Changes in hand grip strength in relation to chronological age and maturity status could improve the predictive power through nonlinear models in schoolchildren.

**Objectives:**

To determine whether nonlinear models provide more accurate and higher predictions of hand grip strength (HGS) in children and adolescents, taking into account chronological age and maturational status.

**Methods:**

A descriptive cross-sectional (correlational) study was designed in schoolchildren aged 6–16 years. The sample selection was non-probabilistic (accidental). A total of 1,048 schoolchildren from 03 public schools were selected (562 males and 482 females). Weight, height and body mass index (BMI) were assessed using standardized anthropometric techniques. The maturational state was estimated by the age at which the maximum velocity of stature (APHV) is reached, using Moore's technique. Handgrip strength (HGS) was evaluated for both hands using a digital dynamometer.

**Results:**

The nonlinear (cubic) relationships showed better explanatory power and fit than the classic linear model in both sexes and hands (HGS). In male schoolchildren, the coefficient of determination (R²) of the nonlinear model was 2% to 3% higher than that of the linear model. Meanwhile, in females the R^2^ was higher from 1%–4%, both for chronological age and maturational state. The cubic model showed a better fit of the SEE (in males it ranged between 3.44 and 5.32 and in females between 3.36 and 3.57), large effect sizes (f² > 0.35), evidencing a greater precision and ability to capture the variability of the HGS in both hands in relation to the classical linear model (in males 4.81–5.55 and in females 3.37–5.08).

**Conclusion:**

The results of the study have shown that the cubic model provides a better explanation and fit of the relationship between chronological age and maturity status with HGS than the classical linear model. It was also determined that fluctuations in HGS are more accurately described by maturity status than by chronological age. These results suggest including nonlinear models and controlling for maturity status. This facilitates the design of interventions according to the different stages of maturational development.

## Introduction

Hand grip strength (HGS) is a task involving maximal isometric grip strength, in which individuals squeeze a grip dynamometer with maximal effort for a short period of time, followed by a relaxation phase of the contracted musculature (1).

The HGS presents several advantages, for example, high predictive value, simplicity in use, ease of the measurement procedure, portability, it is low cost (2) and presents a valid and reliable assessment in both healthy people and people with diseases (3). These characteristics appeal to healthcare professionals to assess isometric strength in children, adolescents, and adults in clinical and epidemiological settings (4). It is often used as a means to predict an individual's health throughout life (5, 6).

In general, the HGS is used as part of a battery of physical fitness tests in schoolchildren related to health or sports performance (7). In addition, this test is used in conjunction with other tests (e.g., flexion and extension of arms on the floor, suspension on a bar, abdominal muscular endurance, among others) (8, 9). These tests together allow describing the level of muscular fitness of schoolchildren in a more complete and detailed manner. They offer valuable information for health promotion and program design in physical education classes and sports training.

It is widely known that muscle power and strength in children and adolescents is related to age, sex, growth level, morphological characteristics (10) and maturity status (11).

In fact, during the period of growth and development in adolescents, there is a significant increase in muscle mass (12). Therefore, monitoring biological maturation is important, as studies in general, highlight that maturity status and absolute strength are strongly associated. Since more mature children outperform less mature children during dynamic and static strength assessments in sporting (13–15) and non-sporting (16, 17) populations.

In general, growth curves play a significant role in understanding and modeling the growth of children and adolescents (18). Thus, during childhood and adolescence physical development is characterized by nonlinear changes (19). Which are influenced by complex biological processes such as skeletal, hormonal and neuromuscular maturation (11).

In this context, HGS in pediatric populations is considered as a functional indicator of health and physical performance. Thus, it does not follow a constant growth pattern, but presents accelerations and plateaus related to chronological age and maturational status that reflect non-linear patterns (20).

Indeed, this information could better capture the actual trajectories of HGS development. Recognizing that growth and strength gain do not occur steadily, but in discrete stages and especially during puberty.

For this reason, monitoring HGS in school children and adolescents according to chronological age and maturity status is fundamental to promote their health, physical development, academic performance, sports, and for the prevention of health problems and injuries (21–23).

In that sense, in recent years several studies have been interested in the use of linear regression models to predict HGS in children and adolescents by chronological age and maturity status (4, 23–26). However, to our knowledge, there are no studies that explicitly state that more complex models, such as quadratic or cubic regressions, can provide more accurate predictions of HGS adjusted for chronological age and maturity status in adolescents.

Therefore, applying nonlinear modeling not only responds to a statistical need, but also to a better biological understanding of human development. This is essential to generate more accurate estimates of HGS and to design intervention strategies adjusted to the maturational profile of each child and adolescent.

In general, this research seeks to fill this gap in the scientific literature. Since nonlinear models could show higher predictive values in relation to the linear model between HGS with chronological age and maturity status, in children and adolescents in a region of moderate altitude in Peru.

It is proposed that the relationship between HGS with chronological age and maturational status in children and adolescents does not follow a constant linear pattern. Rather, it presents a growth trajectory with accelerated changes and decelerations throughout development. Therefore, it is hypothesized that a nonlinear model, specifically a cubic model, will be able to describe and predict this relationship with greater accuracy and statistical fit than a traditional linear model.

Therefore, the aim of the study was to determine whether nonlinear models, particularly the cubic model, offer better predictive and explanatory ability for HGS in children and adolescents. In comparison with linear models, considering chronological age and maturational status as independent variables.

## Materials and methods

### Type of study and sample

A descriptive cross-sectional study (correlational) was designed in schoolchildren aged 6–16 years in a region of moderate altitude in Peru.

The sample selection was non-probabilistic (accidental). This was due to the direct accessibility to the participating school population during the data collection period. A total of 1,048 schoolchildren (562 males and 486 females) from the primary (6–11 years) and secondary (12–16 years) levels of 03 public schools in the city of Arequipa (Peru) were selected. This city is located at 2,320 meters above sea level.

All parents and/or legal guardians of the schoolchildren authorized the voluntary participation of the minors by signing an informed consent form. In addition, each of the children and adolescents received an assent, in which they accepted free and voluntary participation in the study.

Schoolchildren who attended physical education classes and those who completed the anthropometric tests and the HGS evaluation were included in the study. Schoolchildren who did not attend the day of the evaluation and those who were on medical rest were excluded. The study was conducted in accordance with the recommendations of the ethics committee of the Catholic University of Santa Maria (UCSM-096-2022) and the Helsinki declaration for human subjects.

### Techniques and procedures

Anthropometric evaluations and HGS of both hands were carried out at the facilities of each school. The collection process was in charge of 04 physical education teachers, both with extensive experience in measurements and evaluation. A card was elaborated to store the data to be collected. Student data (date of birth, sex and year of study), age, weight, height, HGS of both hands were considered. The data collection process was carried out by sex, first the girls were evaluated, and then the boys were evaluated during physical education classes.

Anthropometric measurements were evaluated following the recommendations of Ross & Marfell-Jones (27). Body weight and height were measured with as little clothing as possible (shorts, t-shirt and no shoes). Weight (kg) was assessed with a a digital scale of Seca GmbH & Co. KG, Germany, with an accuracy of (100 g) and a scale of (0–150 kg). The scale was placed on a flat and stable surface. Height (m) was evaluated using a Seca brand aluminum stadiometer graduated in millimeters with a scale of (0–2.50 m). The head was positioned according to Frankfort's plane (an imaginary line passing from the lower edge of the eye orbit to the upper edge of the ear canal). Body Mass Index (kg/m^2^) was calculated through the formula: [BMI = Weight(kg)/Height(m)^2^].

Truncal height (sitting height) was measured using a wooden bench with a height of 50 cm, with a measurement scale from 0–150 cm, and with an accuracy of 1 mm.

The maturity status (MS) of children and adolescents was assessed using the noninvasive anthropometric techniques proposed by Moore et al. (28). This technique uses chronological age and height. It predicts the years of maximum growth velocity (APHV) using a regression equation for each sex. The equations used to estimate the state of maturity in both sexes have a percentage of explanation (R²) of 89% and a standard error of estimation (SEE) of less than 0.52, which are highly valid and reliable. The equations are:

Females: Maturity status (APHV) = −7.709133 + [0.0042232 × (age × height)].
R2 = 0:898 & SEE = 0:528
Males: Maturity status (APHV) = −7.999994 + [0.0036124 × (age × height)].
R2 = 0:896 & SEE = 0:542

This information allows children and adolescents to be classified into different stages of somatic maturation, facilitating the understanding of their physical development. For example, a negative APHV value represents the time prior to this growth peak. While a positive value indicates the time elapsed after reaching it and the zero value is the time of the growth velocity peak.

The HGS (right and left) was evaluated using a JAMAR brand hydraulic dynamometer (Hydraulic Hand Dynamometer ® Model PC-5030 J1, Fred Sammons, Inc., Burr Ridge, IL: USA). This equipment has an accuracy of 0.1 kg and a scale up to 100 kg/f. The protocol proposed by Richards et al. (29) was used to measure this test. The volunteers were held in a sitting position (seated in a straight-backed chair). One of the evaluators constantly adjusted the dynamometer to the grip size of the equipment according to age and sex. The volunteers performed two attempts with each hand and had a rest between both repetitions a 2 min interval as suggested by Gomez-Campos et al. (6). Prior to the dynamometer measurements, a dress rehearsal was performed with all children and adolescents. Each participant performed a practice run with both hands to familiarize him/her with the evaluation process (three maximum attempts alternating with rest pauses).

### Statistics

The normal distribution of the anthropometric data and the HGS were verified by means of the Kolmogorov–Smirnov test. Descriptive statistics were calculated: mean, standard deviation, and range. Differences between both sexes and both age groups were performed by *t*-test for independent samples.

Linear and nonlinear models (quadratic, cubic and quartic) were applied. Where the best statistical fit according to the adjusted coefficient of determination (adjusted R²), the standard error of estimation (SEE) and the random distribution of the residuals was the cubic polynomial model (third degree) for both sexes, both for age and HGS and maturity status APHV and HGS:

HGS = a + b_1_(age) + b_2_(age)^2^ + b_3_(age)^3^, where: a is the inter-section, b_1_, b_2_ and b_3_ are regression parameters, estimated from the data.
APHV = a + b_1_(APHV) + b_2_(APHV)^2^ + b_3_(APHV)^3^, where: a is the inter-section, b_1_, b_2_ and b_3_ are regression parameters, estimated from the data.

Regression coefficients, adjusted R², as well as effect size (f²) were also calculated for each model, following Cohen's classification. In addition, nested model comparisons were applied using *F*-tests (*F*-test) for each combination of sex, hand tested and predictor variable.

The quartic model was not adopted, as it did not significantly improve the fit and presented risks of overfitting in our data.

The regression analysis was performed separately for each sex. For all cases, *p* < 0.05 was adopted and calculations were performed in Excel spreadsheets, SPSS 18.0 and MedCalc 11.1.0.

## Results

The variables characterizing the schoolchildren studied are shown in Table 1. Males presented greater body weight in relation to females at 15 and 16 years of age and in height from 14–16 years of age (*p* < 0.05). Females showed a more advanced maturity stage in relation to males, reaching APHV at 12 years of age and in males around 14 years of age. Differences in maturity status between both sexes begin to appear from 10 years onwards (*p* < 0.05). In the HGS of both hands, males showed greater strength than their female counterparts. These differences are significant from 14–16 years of age (*p* < 0.05). There were no significant differences in BMI between both sexes and at all ages (*p* > 0.05).

**Table 1:** Anthropometric and HGS indicators of the schoolchildren studied.

Age (years)	n	Weight (kg)		Height (cm)		BMI (kg/m^2^)		MS (APHV)		HGS-R (kg)		HGS-L (kg)	
		X	SD	X	SD	X	SD	X	SD	X	SD	X	SD
Males													
6	36	24.2	5.0	118.2	6.5	17.2	2.0	−5.2	0.2	15.5	3.7	15.8	4.2
7	38	28.2	6.4	123.2	6.7	18.4	2.8	−4.7	0.2	18.6	5.1	19.2	5.6
8	35	29.6	5.6	128.5	5.2	17.8	2.3	−4	0.2	19.3	4.7	20.9	5.3
9	52	35.6	8.6	132.6	8.6	20.0	3.3	−3.4	0.4	23.6	5.7	23.1	5.0
10	72	39.4	9.0	137.3	7.0	20.8	4.1	−2.8	0.3	25.0	6.0	25.3	6.8
11	62	49	9.1	148.6	9.1	21.9	3.8	−1.8	0.4	25.1	9.4	25.8	7.9
12	68	47.5	9.9	152.6	7.9	20.3	3.4	−1.1	0.4	29.1	9.4	29.5	8.3
13	47	53.3	8.9	160.4	7.4	20.6	3.5	−0.2	0.4	42.2	9.7	40.5	9.0
14	50	55.4	8.7	164.1	6.9	20.5	2.5	0.6	0.4	45.3	7.7	45.5	7.2
15	47	61	9.7	165.8	5.1	22.2	3.3	1.3	0.3	42.4	10.2	41.7	8.4
16	55	59.4	6.8	167.7	6.1	21.1	2.1	2	0.4	42.6	9.8	41.3	9.3
Females													
6	25	23.4	3.6	119.5	4.8	16.4	2.1	−4.4	0.2	14.7	2.8	16.0	4.6
7	42	26.3	5.4	123.2	6	17.2	2.4	−3.8	0.3	17.4	4.8	18.0	5.5
8	41	30.1	7.3	127.5	6.9	18.3	3.0	−3.1	0.4	20.1	6.6	20.2	6.3
9	70	33.8	8.6	132.7	7.2	19	3.5	−2.4	0.4	23.0	4.6	22.9	5.4
10	64	40.9	8.9	140.1	8.6	20.7	4.3	−1.5	0.5	24.3	7.1	24.8	7.6
11	47	45.8	8.1	147.4	6.9	20.1	3.1	−0.6	0.4	24.5	6.7	25.0	7.0
12	54	47.5	8.0	151.9	5.5	20.5	3.0	0.3	0.3	28.3	9.3	28.7	9.2
13	34	52	8.6	153.1	5.6	22.1	2.8	1.0	0.4	35.3	7.2	35.6	6.9
14	38	54.4	6.1	154.8*	4.3	22.7	2.1	1.7	0.3	37.8*	7.5	38.1*	7.6
15	38	55.7*	9.0	155.7*	5.1	22.9	3.2	2.6	0.4	34.5*	6.3	34.3*	5.7
16	33	56.1*	9.2	155.3*	5.0	23.3	5.8	3.1	0.4	35.2*	6.8	35.4*	5.9

Legend: X, mean; SD, standard deviation; BMI, body mass index; MS, maturity status; APHV, peak years of growth velocity; HGS-R, right hand grip strength; HGS-L, left hand grip strength.

* Significant difference in relation to males (*p* < 0.05).

Table 2 shows the coefficients of determination (R²), effect size (Cohen's f²), *F* values, SEE and estimated parameters for the linear and cubic models, according to sex, predictor variable [Chronological age and maturity stage (APHV)] and right and left hand.

**Table 2:** Values of the linear and non-linear relationship between age and maturity stage with HGS.

Models	HGS (right)					Parameter estimates				F (*F*-test) nested	HGS (left)					Parameter estimates				F (*F*-test) nested
	R^2^	Cohen's f^2^	F	p	SEE	C	b1	b2	b3		R^2^	Cohen's f^2^	F	p	SEE	C	b1	b2	b3	
Age (years)																				
Males																				
Linear	0.76	1.37	1,746.90	0.000	5.37	−17,79	3,234	–	–	–	0.76	1.37	1,478.88	0.000	5.55	−16,61	3,127	–	–	–
Cubic	0.79	1.66	67.876	0.000	5.06	48,01	−12,74	1,215	−0,029	38,29*	0.78	1.66	8.279	0.000	5.32	6,193	−0,99	0,173	.000	38,29*
Females																				
Linear	0.65	0.73	87.585	0.000	3.64	−4,054	1,73	–	–	–	0.7	0.73	94.203	0.000	3.72	−5,259	1,848	–	–	
Cubic	0.68	0.86	32.492	0.000	3.57	28,74	−8,317	0,966	−0,029	21,09*	0.74	0.86	367.39	0.000	3.52	32,965	−9,931	1,139	−0,04	21,09*
MS (APHV)																				
Males																				
Linear	0.81	1.91	2,309.60	0.000	4.81	27,22	4,358	–	–	–	0.79	1.91	1,855.80	0.000	5.08	26,825	4,222	–	–	–
Cubic	0.83	2.21	863.40	0.000	4.59	26,48	5,244	0,192	−0,025	38,40*	0.82	2.21	684.70	0.000	3.44	25,782	5,063	0,252	−0,01	38,40*
Females																				
Linear	0.70	0.96	1,098.30	0.000	3.37	17,34	2,254	–	–	–	0.75	0.96	1,175.21	0.000	4.88	17,593	2,405	–	–	–
Cubic	0.72	1.08	378.17	0.000	3.36	17,92	2,456	−0,117	−0,027	20,00*	0.76	1.08	416.62	0.000	3.36	18,444	2,675	−0,16	−0,04	20,00*

Legend: SEE, standard error of estimation; APHV, peak years of growth velocity; HGS, hand grip strength; SEE, standard error of estimation; C, Constant; f^2^, effect size.

* Significant difference in relation to the linear model (*p* < 0.001).

In all cases, the cubic models presented a higher R² (in males, >2%–3% and in females higher >1%–4%), a higher effect size (f²) than the linear model (in all cases, the effect size is large >0.35). Even in the cubic model, lower values of SEE were observed than in the classic linear model.

Table 2 also shows the nested model comparison analysis (*F*-test) to determine the statistical significance of the improvement of the cubic model over the classical linear model (by chronological age, and by APHV maturity stage). In all cases, the inclusion of additional polynomial terms (Age² and Age³, and APHV^2^ and APHV^3^) significantly increased the variance explained and reduced the residual error. For males, *F*(2, 536) = 38.29 (*p* < 0.001) was obtained for chronological age for both hands and *F*(2, 536) = 38.40 (*p* < 0.001) for APHV maturity status. For females, *F*(2, 450) = 21.09 (*p* < 0.001) for chronological age and for both hands and *F*(2, 450) = 20.0 (*p* < 0.001) for APHV for both hands. These results confirm that the improvement in the cubic model fit is statistically significant and not due to chance.

**Figure 1:**
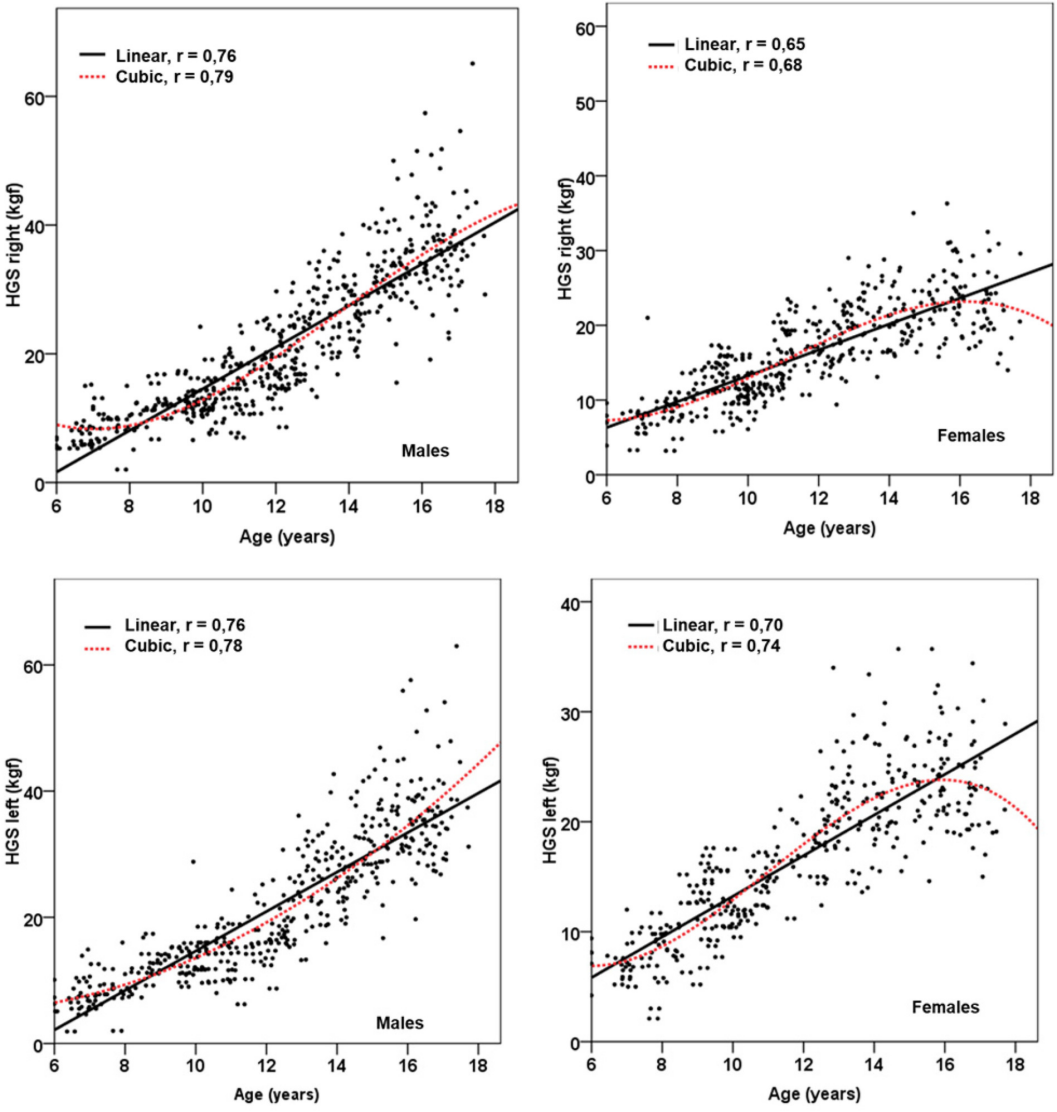
Linear and non-linear (cubic) relationship between chronological age and HGS of both hands and in both sexes.

**Figure 2:**
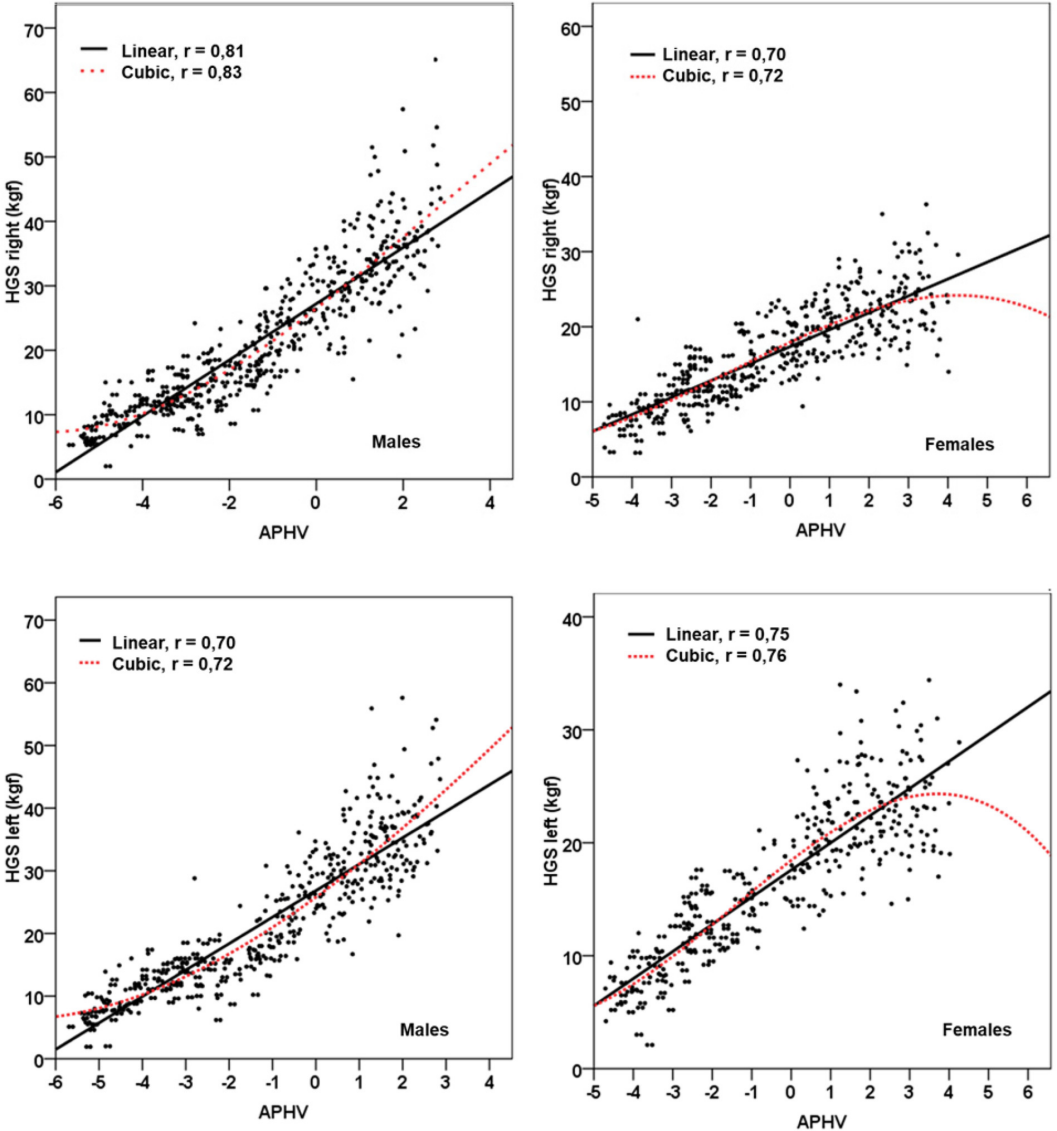
Linear and non-linear (cubic) relationship between maturity status and HGS of both hands and in both sexes.

In summary, the cubic model demonstrated a superior fit compared to the traditional linear model, offering greater accuracy and predictive capability. This is clearly illustrated in Figures 1, 2.

## Discussion

The results of this study have shown that the relationship between HGS with chronological age and maturity status (APHV) in children and adolescents does not follow a strictly linear pattern. In fact, after data analysis, the cubic model presented a higher coefficient of determination (R²), large effect sizes (Cohen's f²) and a lower SEE compared to the classical linear model.

In addition, after the application of the *F* tests for nested comparisons, they allowed us to confirm that there was a better fit in the cubic polynomial models. They were also significant in both hands and sexes (*p* < 0.001), both for chronological age and APHV maturity status.

Overall, these findings support that the evolution of HGS in child and adolescent population follows nonlinear patterns linked to the biological changes of physical growth and pubertal maturation. This is in agreement with what has been proposed by some studies, where the trajectory of growth and somatic maturation present nonlinear dynamics (18, 19). This confirms the hypothesis put forward in our study.

Indeed, the physical growth curve plays an important role in understanding growth trajectories over time and in examining the mathematical relationship between the outcome variable and time (18). Therefore, the nonlinear (cubic) model allows to evidence these inflection points (30), reflecting more clearly and realistically the complexity of the relationship between age and maturity with the evolution of HGS. Whereas the classical linear model only assumes a continuous and uniform relationship between the predictor and criterion variable. Therefore, it does not allow the identification of possible break points or abrupt changes in the slope of the relationship.

In sum, the results of this study showed that the behavior of HGS in children and adolescents does not follow a linear pattern, unlike what has been reported by some previous studies (4, 17, 26). This suggests that the evolution of HGS responds to growth and maturation processes that do not conform to linear trajectories, but also present more complex changes that require the analysis of specific nonlinear models to adequately describe this pattern.

In this sense, the behavior observed in schoolchildren of both sexes, both by chronological age and by maturity status aligned with HGS, allows us to highlight these complex changes. For example, in males at early ages (6–8 years) and/or prepuberty (−5APHV and −4PHV), HGS remains relatively stable, followed by a slow increase from age 9–13 years and/or (−3APHV to 0APHV). Subsequently, at age 14 years and/or (+1PHV) there is a rapid increase in HGS until about age 17 years. In females at early ages (6–8 years) and/or prepubertal (−5APHV and −4PHV), HGS remains relatively stable as in males. However, after 9–14 years and/or (−3APHV to +1PHV), HGS increases rapidly until reaching a plateau at 15, 16, and 17 years and/or (+2APHV, +3APHV, and +4APHV).

Consequently, the greatest increase in HGS in both hands is evidenced precisely at the OAPHV level for both sexes (at approximately 12 years in females and 14 years in males). The use of the cubic model made it possible to reflect the natural variability of HGS, offering a tighter representation of the fluctuations linked to age and maturation status. This is corroborated in the literature (11, 31).

On the other hand, it was also observed that models that include maturity status (APHV) as a predictor variable explain a greater proportion of the variance of HGS compared to chronological age. This reinforces the importance of considering biological indicators of maturity in the assessment of physical development of children and adolescents in general.

These findings allow highlighting that maturity status values determined by APHV reflect higher values of explanation, lower values of SEE and large effect sizes relative to chronological age in both sexes. This also demonstrates that HGS analysis should be analyzed not only by chronological age, but also by maturity status, as highlighted in the literature (11).

These results suggest that the application of cubic models allows to better capture fluctuations in HGS. Especially when associated with changes in maturity status. This could improve accuracy in the assessment of physical development in adolescent schoolchildren.

Therefore, the assessment and monitoring of maturity status can provide valuable information to practitioners and researchers in this area to better evaluate and compare youngsters of similar chronological age during a period in which biological age can vary by up to approximately five years (11, 32). In that sense, morphological and body composition changes during the growth and maturation period are determinant for strength development and, consequently, for performance in motor tasks such as running, jumping, and throwing (33).

Consequently, controlling for interindividual differences is important when monitoring maturity status in youth sport, for talent development, and for categorizing physical activity levels in schoolchildren (34). As well as for designing conditioning and training programs for young athletes and schoolchildren (35, 36).

Therefore, taking into account maturity status control along with chronological age is essential for effective assessment and planning of physical development in school-aged children and especially HGS. This not only improves the accuracy of assessment, but also contributes to the design of training programs that optimize performance and minimize the risk of injury in school-aged children and adolescents.

The information provided in this study can serve as a reference for health and physical education professionals, facilitating the interpretation of HGS patterns in growing and maturing schoolchildren. Likewise, the findings of the study may be useful in screening programs, design of interventions adapted by biological maturity and promotion of physical activity based on individual development.

The study has some limitations that should be acknowledged. For example, it was not possible to evaluate other physical tests of muscle strength such as horizontal jump, arm strength (medicine ball throwing, among others). In addition, it is necessary to control for fat-free mass in future studies. Since this information could clarify the relationship between body composition and performance in physical fitness tests in the schoolchildren studied. In addition, the study design was cross-sectional and the sample selection non-probabilistic. This limits the ability to establish causal relationships and the generalization of the results to other sociocultural contexts.

It also has some strengths, for example, it is one of the first studies conducted in Peru at moderate altitude. This can serve as a baseline for future comparisons and monitoring of secular trends. The study contributes to the scientific evidence with updated data for a specific population (moderate altitude Peru), where the available information is scarce and limited, thus contributing to fill an important gap in the literature. The use of nonlinear models made it possible to capture complex patterns of development that would not be evident with traditional approaches. Thus, the nonlinear approach constitutes a methodological novelty that allows a more accurate description of physical changes during growth and biological maturation.

As future guidelines, it is suggested that studies in general use or apply longitudinal designs to confirm these findings. In addition, they should include nonlinear models in their analyses to more accurately describe the evolution of HGS and other physical tests related to muscle strength in pediatric populations and especially in other social, cultural, and geographic contexts.

## Conclusions

The results of this study show that the cubic model provides a better fit and a more accurate explanation of the relationship between chronological age, maturity status and hand grip strength (HGS) compared to the linear model. An initial phase of acceleration in HGS growth is observed, followed by a stabilization stage and, in some cases, a further acceleration in later stages. Males experience a more pronounced increase during late maturation, whereas in females the growth is more progressive. In addition, it was found that fluctuations in HGS are more accurately described when maturity status is considered rather than chronological age. This highlights the relevance of incorporating biological indicators in the assessment of muscle development. These findings suggest including nonlinear models and controlling for maturity status. This facilitates the design of interventions according to the different stages of maturational development.

## Data Availability

The raw data supporting the conclusions of this article will be made available by the authors, without undue reservation.
